# Laparoscopically harvested omental flap for immediate breast reconstruction: a retrospective single-center study of 300 cases

**DOI:** 10.1186/s12957-024-03377-7

**Published:** 2024-04-16

**Authors:** Hao Liu, Xiao He, Li Li, Neng-Bin Wan

**Affiliations:** The Second Department of Breast Surgery, Hunan Cancer Hospital and The Affiliated Cancer Hospital of Xiangya School of Medicine, Central South University, Changsha, 410013 Hunan China

**Keywords:** Breast reconstruction, Omental flap, Laparoscopy, Mastectomy, Oncoplastic surgery

## Abstract

**Background:**

The laparoscopically harvested omental flap (LHOF) has been used in partial or total breast reconstruction, but most studies on LHOF were case reports or small case series. However, the clinical feasibility and oncological safety of LHOF in oncoplastic breast surgery remains controversial. This study reported our experience applying LHOF for immediate breast reconstruction.

**Methods:**

Between June 2018 and March 2022, 300 patients underwent oncoplastic breast surgery using LHOF at our institution. Their clinicopathological data, complications, cosmetic outcomes, and oncologic outcomes were evaluated.

**Results:**

All patients underwent total breast reconstruction using LHOF after nipple-sparing mastectomy. The median operation time was 230 min (ranging from 155 to 375 min). The median operation time for harvesting the omental flap was 55 min (ranging from 40 to 105 min). The success rate of the laparoscopically harvested pedicled omental flap was over 99.0%. Median blood loss was 70 ml, ranging from 40 to 150 ml. The volume of the flap was insufficient in 102 patients (34.0%). The overall complication rate was 12.3%. Subcutaneous fluid in the breast area (7%) was the most common reconstruction-associated complication, but most cases were relieved spontaneously. The incidence rate of omental flap necrosis was 3.3%. LHOF-associated complications occurred in two cases, including one case of incisional hernia and one case of vascular injury. Cosmetic outcomes were satisfactory in 95.1% of patients on a four-point scale by three-panel assessment and 97.2% using the BCCT.core software. Two local and one systemic recurrence were observed during a median follow-up period of 32 months.

**Conclusions:**

The LHOF for immediate breast reconstruction is a safe and feasible method that involves minimal donor-site morbidity, satisfactory cosmetic outcomes, and promising oncologic safety.

## Background

Breast cancer is the most common malignancy in women, which tends to affect younger patients [1]. With the diversification in breast reconstruction surgery techniques, the cosmetic expectations of breast tumor patients have increased. For breast cancer patients who are ineligible for breast-conserving surgery (BCS), mastectomy procedures such as nipple-sparing mastectomy (NSM) followed by immediate breast reconstruction with autologous tissue could be a viable alternative method. The transverse rectus abdominis myocutaneous (TRAM) flap and latissimus dorsi (LD) myocutaneous flap are widely used autologous flaps in oncoplastic breast surgery [2]. Nevertheless, these techniques impose several disadvantages, including donor-site morbidities and deformity, volume loss, and an inevitable large donor-site scar [3, 4].

The use of the pedicled omental flap for immediate breast reconstruction was first described by Kiricuta in 1963 [5]. The omental flap is a unique type of flap due to its soft texture, anti-infective properties, and regenerative properties during ischemia [6]. Initially, the use of the omental flap in breast reconstruction was limited due to complications associated with harvesting the flap through laparotomy [7]. In the early 2000s, advances in laparoscopic technology and surgical skills enabled the harvesting of the omental flap through minimally invasive procedures [8–10]. Some researchers reported their experience of immediate breast reconstruction using laparoscopically harvested omental flap (LHOF) [11–14]. The results indicated that using LHOF for breast reconstruction led to fewer complications. Eastern Asian surgeons reported that LHOF was a feasible option for partial or total breast reconstruction [15–17]. Western researchers also applied LHOF reconstruction in certain breast cancer patients [18, 19], although the majority of reports only included a small sample size. Still, some studies suggested that breast reconstruction using the omental flap had a high incidence of digestive complications, e.g., epigastric discomfort, persistent epigastric pain, bowel obstruction and colectomy and a high risk of oncological recurrence, thereby restricting its application to the breast reconstruction of huge defects only [20, 21]. There has been significant concern regarding the oncological safety of using the omental flap in breast reconstruction.

This study reports our experience using LHOF for immediate breast reconstruction, detailing the technique, surgical complications, cosmetic results, and oncologic outcomes.

## Methods

### Patients

**Fig. 1:**
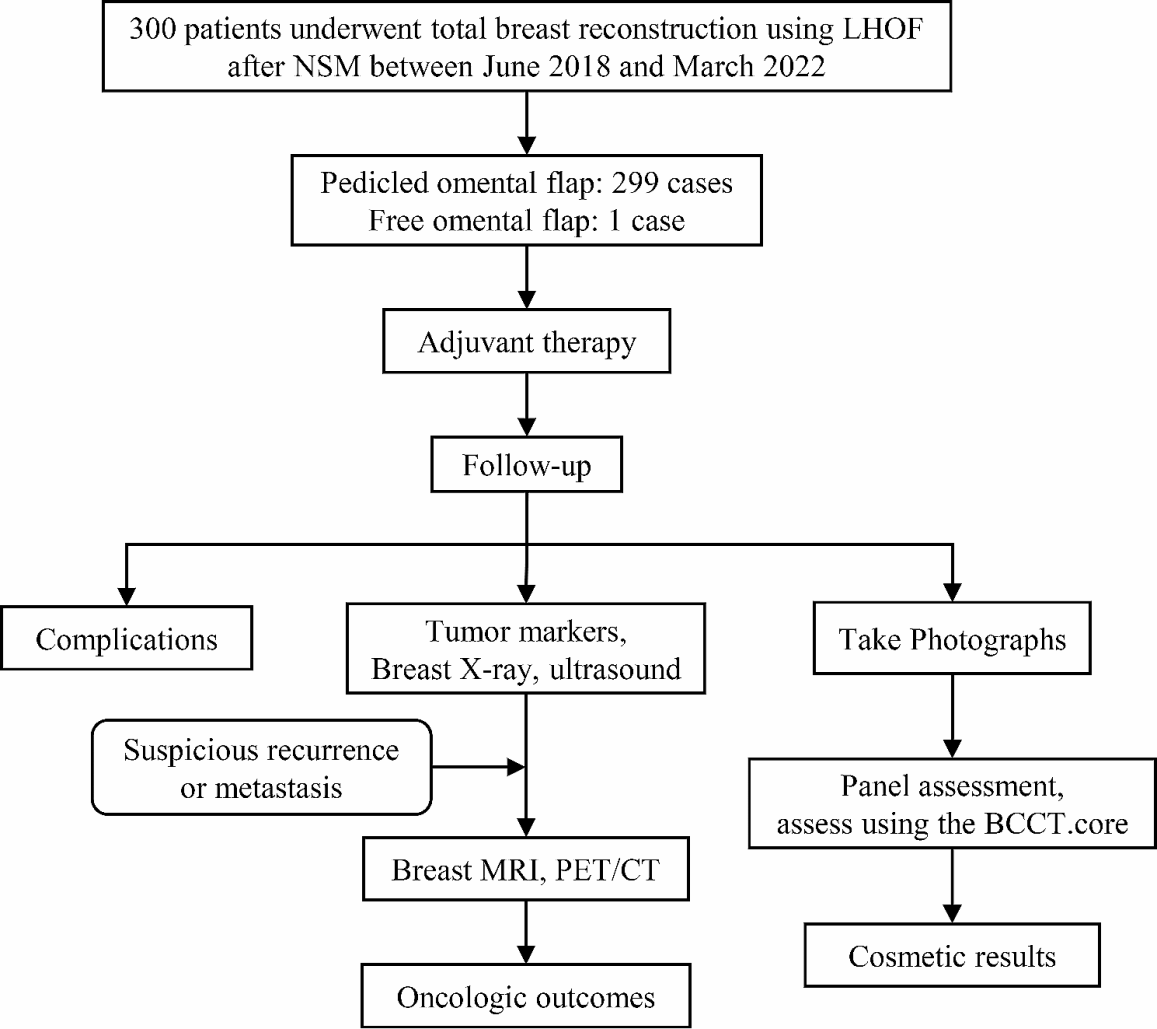
The progress summary of this study

Between June 2018 and March 2022, a total of 300 patients with breast tumors underwent NSM accompanied by immediate breast reconstruction using LHOF at a tertiary cancer center. Preoperative breast ultrasound, mammography, and magnetic resonance imaging (MRI) were performed to evaluate the tumor in all patients. A biopsy was performed to obtain a histopathological diagnosis prior to surgery. The distance between the tumor and the nipple should exceed 20.0 mm, with no invasion of the local skin. Negative distant metastasis was confirmed preoperatively through chest and abdomen computerized tomography (CT) as well as a bone scan. Patients receiving neoadjuvant therapy were included in the study. In contrast, patients with a history of intra-abdominal malignancy or upper abdominal laparotomy were excluded. The medical records were reviewed to obtain the patients’ clinicopathological characteristics, operation duration, length of hospital stay, and complications. The present study was approved by the Ethics Committee of our institution, and all patients provided written informed consent. The progress summary of this study was shown in Fig. 1.

### Surgical technique

#### NSM and subcutaneous tunneling

**Fig. 2:**
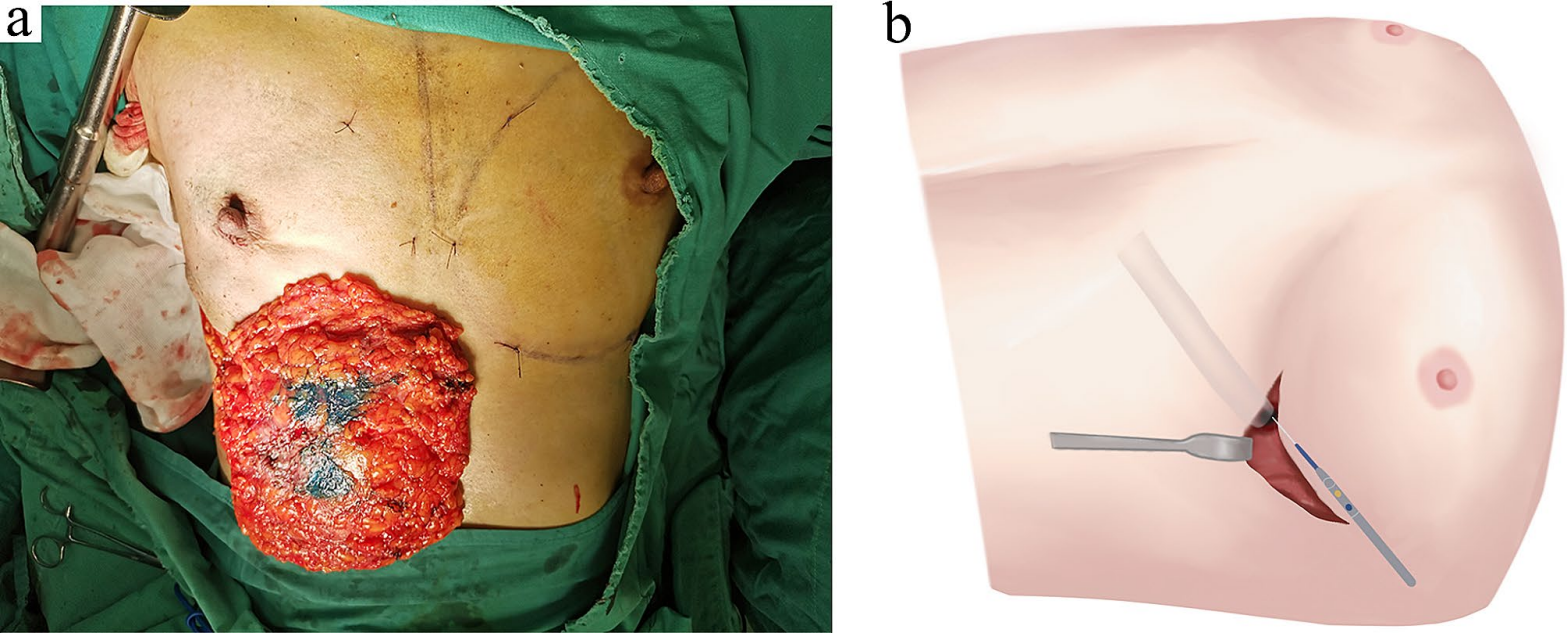
NSM and the scheme for subcutaneous tunneling. (**a**) Patient’s mammary tissue sample following NSM. (**b**) The diagram of subcutaneous tunnel. NSM: nipple-sparing mastectomy

The surgery was performed under general anesthesia, and the patient was placed in the supine position with the bilateral arms abducted at 90°. Regardless of the tumor location, an inframammary fold incision was typically used to perform the NSM. In most cases, the serratus anterior fascia was preserved. In cases with a superficial tumor, a larger amount of subcutaneous fat tissue was removed above the tumor to ensure a negative margin of local skin. Subsequently, the core glandular tissue behind the nipple was resected along with the entire mammary tissue. The nipple was inverted inward for the clean excision of all glandular tissue. In addition, the under surface of Nipple areola complex (NAC) tissue was sent for the frozen section analysis in all patients by taking multiple point specimens from coring out the nipple. Sentinel lymph node biopsy and axillary lymph node dissection were performed according to axillary lymph node involvement (Fig. 2a). After completing the NSM, a subcutaneous tunnel of a width of two fingers was created. The direction was perpendicular to the costal arc and the shortest distance from the inframammary fold incision. The scheme of subcutaneous tunnel was shown in Fig. 2b.

#### LHOF

**Fig. 3:**
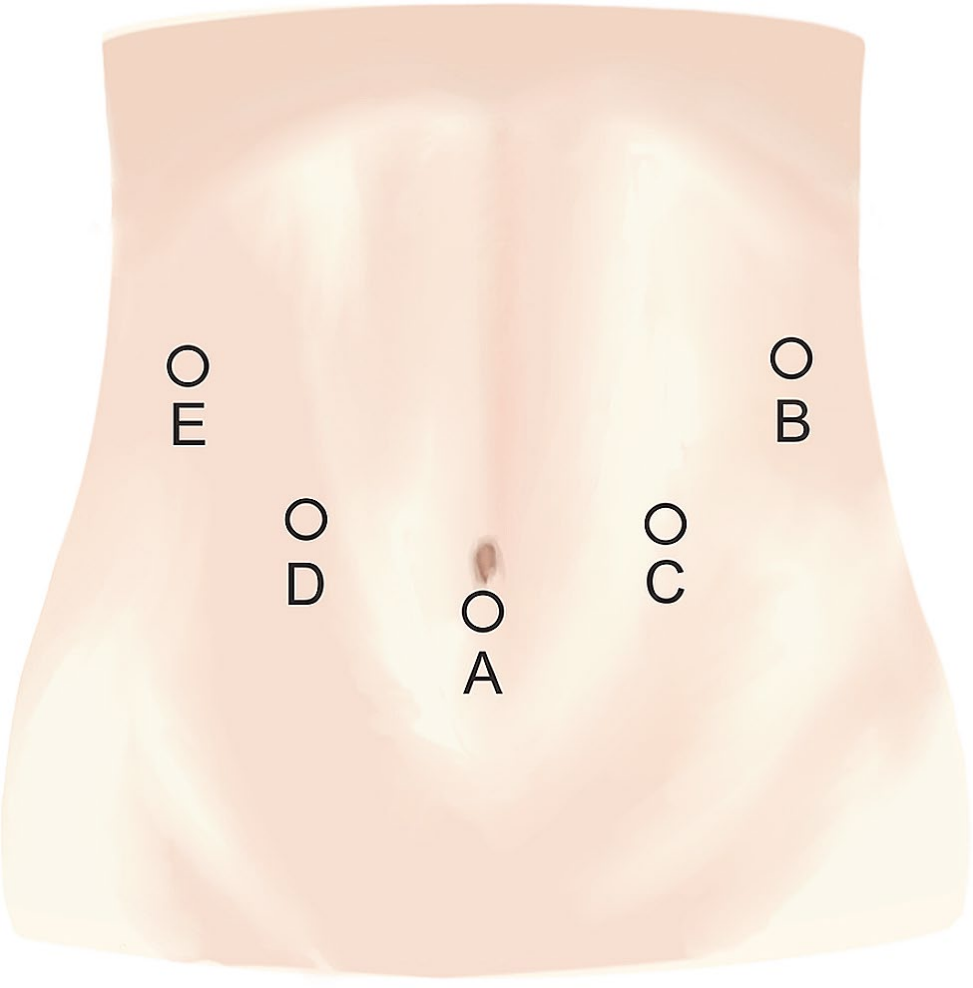
The schematic of port arrangement for LHOF. Port **A**: a 10-mm port placed below the umbilicus for camera; Port **B**: a 10-mm port was placed at the anterior axillary line for main manipulation; Port **C**: 5-mm port was placed at the left midclavicular line; Port **D** and **E**: two 5-mm ports for assistant were palced at the right midclavicular line and anterior axillary line, respectively. LHOF: laparoscopically harvested omental flap

A camera port (10 mm, 30°) was inserted beneath the umbilicus, with the surgeon positioned on the patient’s left side. The intra-abdominal pressure of the pneumoperitoneum was maintained at 12 mmHg. A 10-mm operating port and a 5-mm assisting port were inserted into the patient’s left abdominal wall. The operating port was inserted through the left upper quadrant at the anterior axillary line, while the assisting port was inserted above the umbilicus level through the left midclavicular line. Two 5-mm ports were inserted on the right side to allow the assistant to use instruments for traction and exposure. The scheme of port arrangement was shown in Fig. 3. Firstly, a laparoscopic inspection of the internal abdominal organs was performed to assess the adhesion, size, and vascular supply of the omentum. The omental flap was then harvested.

**Fig. 4:**
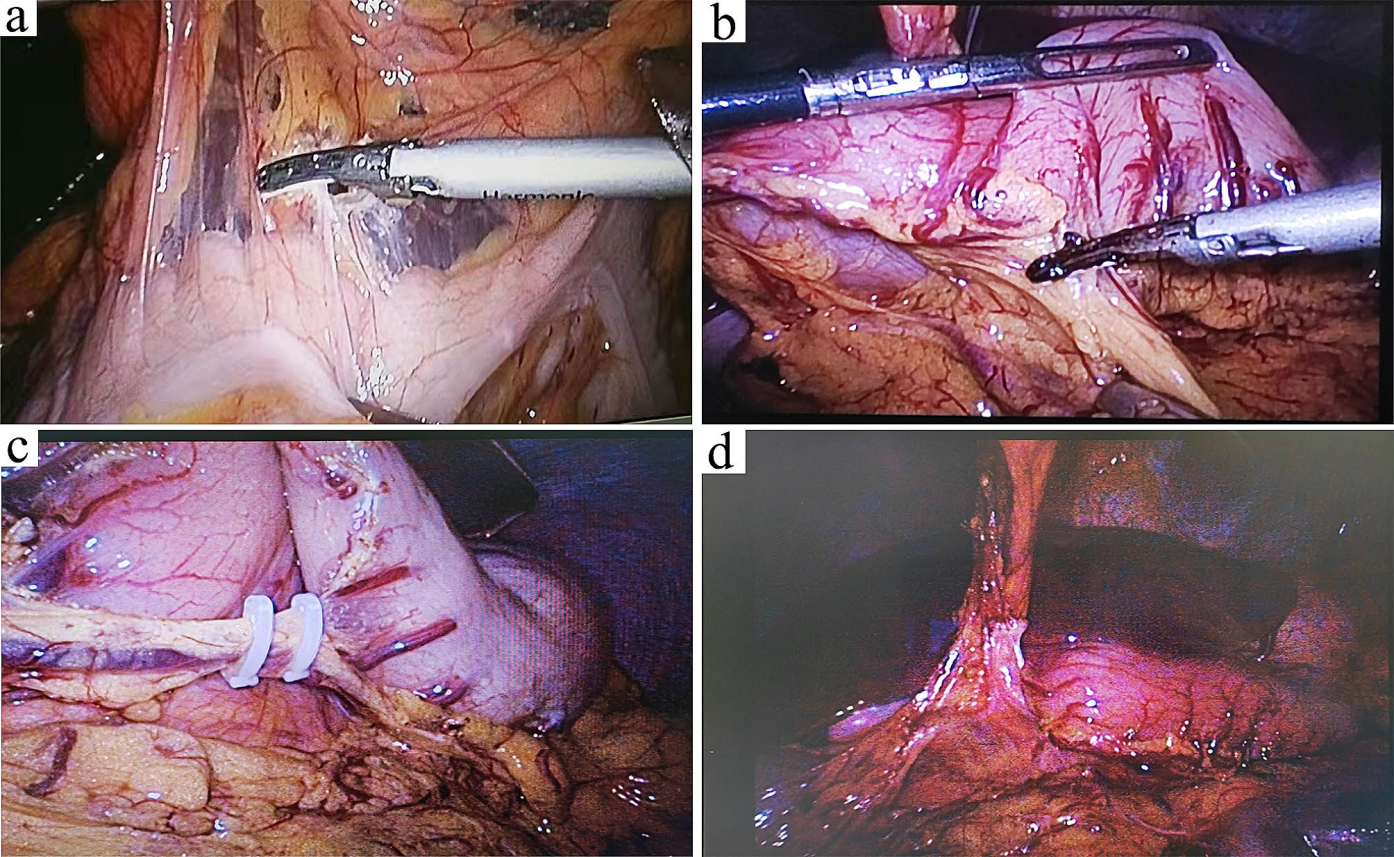
Laparoscopic harvest of the pedicled omental flap. (**a**) The omentum was dissected from the transverse colon. (**b**) The omentum was separated from the stomach wall. **c** The left gastroepiploic vessels were identified and ligated. (**d**) The root of the right gastroepiploic vessels was preserved as the pedicle of the omental flap

**Fig. 5:**
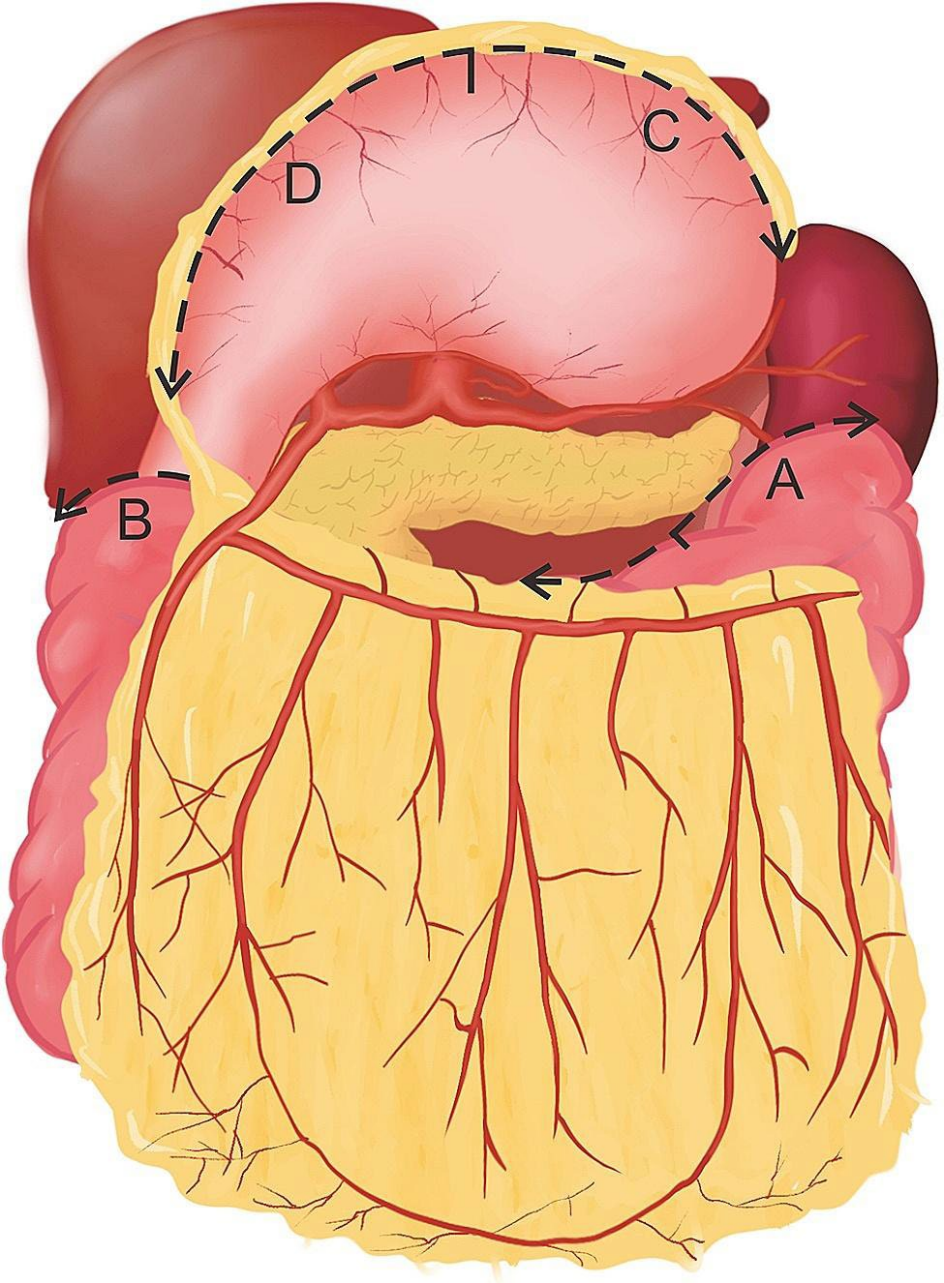
The surgical scheme of the harvesting pedicled omental flap. (**A**) Dissection along the transverse colon from the midpoint to the splenic flexure; (**B**) Dissection along the transverse colon from the midpoint to the hepatic flexure; (**C**) Dissection along the greater curvature of the stomach from the midpoint to the left; (**D**) Dissection along the greater curvature of the stomach from the midpoint to the pyloric ring

The omentum was dissected from the midpoint of the transverse colon to the left at approximately 1 cm above the transverse colon (Fig. 4a). Visualization of the posterior gastric wall indicated that the lesser sac was reached, confirming the correct anatomical level. Dissection was continued to the left, and the omentum was transected around the spleen. The omentum was further dissected towards the right side, along the transverse colon, until reaching the hepatic flexure. Furthermore, starting from the midpoint of the greater curvature of the stomach, the omentum was dissected to the left. To prevent injury to the gastroepiploic hemal arch, the gastric branches were dissected as close to the stomach wall as possible, up to the main trunk of the left gastroepiploic vessels (Fig. 4b). The left gastroepiploic vessels was then ligated by a titanium clip and severed (Fig. 4c). Subsequently, the omentum was further dissected to the right until passing the pyloric ring. The roots of the right gastroepiploic vein and artery were preserved as the pedicle of the omental flap (Fig. 4d). All dissections were performed using the Harmonic Scalpel. The surgical scheme of LHOF was shown in Fig. 5.

**Fig. 6:**
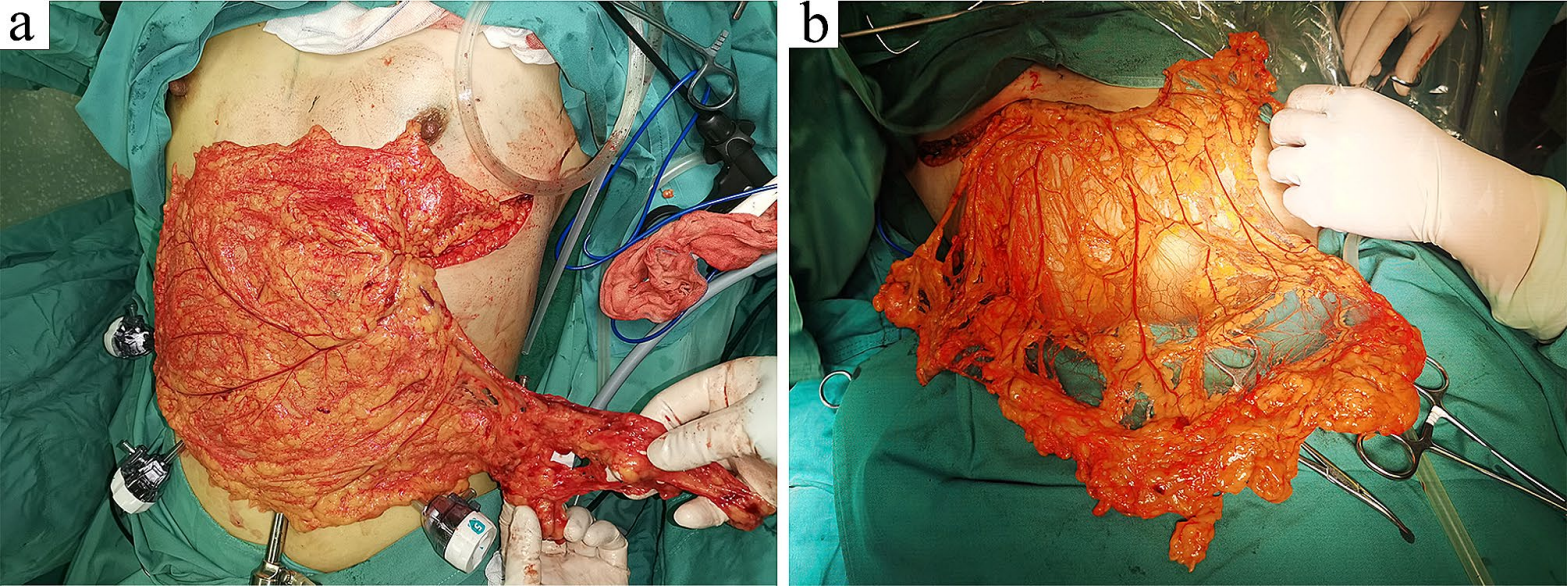
A pedicled omental flap was extracted through the subcutaneous tunnel. (**a**) A well-vascularized omental flap with abundant adipose tissue. (**b**) A thin pedicled omental flap

Under laparoscopic vision, an incision was made on the white line to communicate with the subcutaneous tunnel. Sponge forceps were inserted into the abdominal cavity through the tunnel to pull out the pedicled omental flap. The pedicle was kept free of tension and without twisting. After re-inspecting the abdominal cavity, the trocars were withdrawn, and the gas was released. The blood supply and color of the omental flap were carefully examined (Fig. 6).

#### Breast reconstruction

The omental flap was unfolded and placed on the surface of the pectoralis major muscle to fill the space of the breast defect. If the volume of the omental flap was insufficient, a prosthetic implant was inserted. Saline mixed with iodophor was used to sterilize the breast space. The implant was placed behind the pectoralis major muscle. The fascia of the serratus anterior muscle was separated and a pocket was created by combining it with the pectoralis major muscle to enclose the prosthetic implant. Several interrupted sutures were tied between the fascia and the muscle to immobilize the implant, and the omental flap was then unfolded to fully cover the implant and the pectoralis major muscle. The outer orifice of the subcutaneous tunnel was closed using interrupted sutures. Meanwhile, a space of approximately 5 mm around the pedicle was left to avoid compression to the pedicle. The omental flap was not fixed to the chest wall. The patient’s position was adjusted to a semi-recumbent position of 45 degrees, and the reconstructed breast was shaped according to the shape of the contralateral breast. One drainage tube was placed in the lateral region of the breast. In cases requiring axillary lymph node dissection, another drainage tube was placed in the axilla. The incision was closed with interrupted intradermal absorbable sutures.

#### Follow-up

All patients were followed up every 3 months after the operation for one year, and then at six-month intervals thereafter. Complications, cosmetic outcomes, and oncologic results were evaluated. Photographs were taken from three different angles before the surgery, and postoperative pictures were taken at each follow-up using the same method. The cosmetic outcomes were assessed 1 year after surgery using a 4-point scale by three professional physicians [22]. The reconstructed breast was scored as “Excellent”, “Good”, “Fair”, and “Poor” by comparing it with the untreated breast. “Excellent” and “Good” were considered satisfactory. The Breast Cancer Conservative Treatment (BCCT.core; Breast Research Group, INSEC Porto, the University of Porto) software was also used to assess the cosmetic outcomes. The BCCT.core software automatically evaluates cosmetic results by scanning patient pictures. The software classifies the cosmetic results into four levels (excellent, good, fair, and poor) based on symmetry, skin color, and surgical scar [23]. Moreover, the oncologic outcomes were assessed by mammography and breast ultrasound. Distant metastasis was investigated by tumor marker examinations, MRI and/or 18 F-fluorodeoxyglucose positron emission tomography/computed tomography (PET/CT).

## Results

### Clinicopathological characteristics

From June 2018 to March 2022, a total of 300 patients underwent total breast reconstruction using LHOF. All omental flaps were harvested laparoscopically without conversion to laparotomy. The median age was 41 years old (ranging from 33 to 65 years), the mean body mass index was 22.5 kg/m^2^ (ranging from 16.3 to 32.6). Forty-seven patients (15.7%) had a history of abdominal surgery, and nearly 80% of the patients were in the T1 and T2 stages. 81% (243/300) of patients were diagnosed with invasive ductal breast carcinoma. Of the 293 breast cancer patients, 75 (25%) received preoperative neoadjuvant chemotherapy. Postoperative radiotherapy was administered to 22.3% of the patients. The median follow-up time was 32 months, ranging from 10 to 55 months (Table 1).

**Table 1:** Patient characteristics

Variables	*n* (range or ratio)
Age, years, median (range)	41 (33 to 65 )
BMI, kg/m^2^, mean (range)	22.5 (16.3 to 32.6)
Comorbidities	
Diabetes	28 (9.3%)
Hypertension	36 (12.0%)
Ischemic heart disease	14 (4.7%)
Breast cup size	
≤A	113 (37.7%)
B	139 (46.3%)
≥C	48 (16.0%)
Tumor size, cm, median (range)	2.6 (1.5 to 5.2 )
Tumor location, n (%)	
Outer upper quadrant	96 (32.0%)
Outer lower quadrant	86 (28.7%)
Inner upper quadrant	64 (21.3%)
Inner lower quadrant	54 (18.0%)
T stage, n (%)	
pTis	50 (16.7%)
pT1	102 (34.0%)
pT2	132 (44.0%)
pT3	9 (3.0%)
NA	7 (2.3%)
N stage, n (%)	
pN0	216 (73.7%)
pN+	77 (26.3%)
Histopathological type, n (%)	
Invasive ductal carcinoma	243 (81.0%)
Intraductal carcinoma in situ	50 (16.7%)
Phyllodes tumor	7 (2.3%)
Previous abdominal surgeries, n (%)	
Cesarean section	17 (5.6%)
Laparoscopic cholecystectomy	12 (4.0%)
Appendectomy	11 (3.7%)
Hysterectomy and/or oophorectomy	3 (1.0%)
Others	4 (1.3%)
Neoadjuvant chemotherapy, n (%)	75 (25.0%)
Postoperative radiotherapy, n (%)	67 (22.3%)
Follow-up periods, months, median (range)	32 (10 to 55 )

BMI, body mass index

### Surgical outcomes

The median total operative time was 230 min (range: 155 to 375 min). The median time to harvest the omental flap was 55 min (range: 40 to 105 min). Mild adhesions were observed between the omentum and the abdominal wall in 38 patients who had a history of abdominal surgery. Expectedly, the omental adhesion was easily separated using an ultrasonic scalpel. In 34% (102/300) of the patients, the volume of the omental flap was insufficient, and implants had to be used. The median volume of the implant was 125 mL (range: 100 to 210 mL). In addition, the blood loss attributable to LHOF was negligible. All patients were allowed to drink, eat, and walk on the day after surgery. The median duration until drainage tube removal was 6 days. Patients were discharged at a median of 7 days after surgery (Table 2).

**Table 2:** Surgical outcomes

Variables	*n* (range or ratio)
Type of flap, n (%)	
Pedicled omental flap	299 (99.7%)
Free omental flap	1 (0.3%)
Total operation time, min, median (range)	230 (155 to 375)
Time of omentum harvest, min, median (range)	55 (40 to 105)
Conversion to laparotomy	0 (0)
Blood loss, ml, median (range)	70 (40 to 150)
Blood loss of harvesting omentum, ml, median (range)	NA
Prosthetic implant, n (%)	102 (34%)
Volume of prosthesis, ml, median (range)	125 (100 to 210)
Duration of drainage, days, median (range)	6 (3 to 10)
Postoperative hospital stay, days, median (range)	7 (4 to 15)

NA: not applicable

### Complications and oncologic outcomes

The incidence rate of overall complications was 12.3% (Table 3). Two patients experienced complications related to laparoscopy. In one case, the main trunk of the right gastroepiploic vessel was accidentally injured. The omentum was salvaged by establishing an anastomosis between the right gastroepiploic vessels and the thoracodorsal vessels. During the operation, the blood supply of the free omental flap was observed under direct vision to ensure successful reconstruction of circulation. Color Doppler ultrasonography was used to monitor the blood flow of the free omental flap after surgery. One patient developed a ventral hernia and was treated with herniorrhaphy. Several patients complained with transient mild epigastric discomfort, but no persistant epigastric pain, bowel obstruction or bowel perforation were observed.

**Table 3:** Complications and oncologic outcomes

Variables	*n* (%)
Total complications	37 (12.3%)
Complications associated with laparoscopy	
Vascular injury	1 (0.3%)
Ventral hernia	1 (0.3%)
Complications associated with the breast	
Subcutaneous fluid in breast area	21 (7.0%)
Partial omental flap necrosis	10 (3.3%)
Skin flap necrosis	0
Hemorrhage	4 (1.3%)
Infection	0
Oncologic outcomes	
Local recurrence	2 (0.7%)
Distant metastasis	1 (0.3%)

Furthermore, thirty-five patients experienced reconstruction-associated complications. Subcutaneous fluid accumulation in the breast area was observed in 7% (21/300) of patients. The most common complication in this study was subcutaneous fluid, which included hematoma or seroma. Notably, subcutaneous fluid accumulation was more common in patients with large breasts, which may be attributed to a drooping breast, thereby compressing the pedicle of the omental flap. The subcutaneous fluid was mostly alleviated by prolonged drainage. Needle aspiration guided by ultrasound was only necessary when the fluid volume was significant. Ten patients (3.3%) experienced partial necrosis of the omental flap. The partial necrosis was treated conservatively with prolonged drainage and debridement but unfortunately resulted in volume loss of the omental flap. None of the patients experienced skin flap necrosis, necrosis of the nipple-areola complex, or wound infection.

Throughout the entire follow-up period, two cases had local recurrences in the skin flap, and one case had liver metastasis. Both patients with local recurrence were treated with extended local excision and radiotherapy. At 1.5 years postoperatively, the patient’s serum carcinoembryonal antigen (CEA), cancer antigen (CA) 15 − 3 and CA125 levels were elevated. Abdominal ultrasonography revealed a well-defined mass in the liver. PET/CT showed a solitary liver metastasis in the liver parenchyma. Liver biopsy was conducted, and the histological findings were breast cancer metastasis. The patient who had liver metastasis was treated with systemic therapy.

### Cosmetic outcomes

**Fig. 7:**
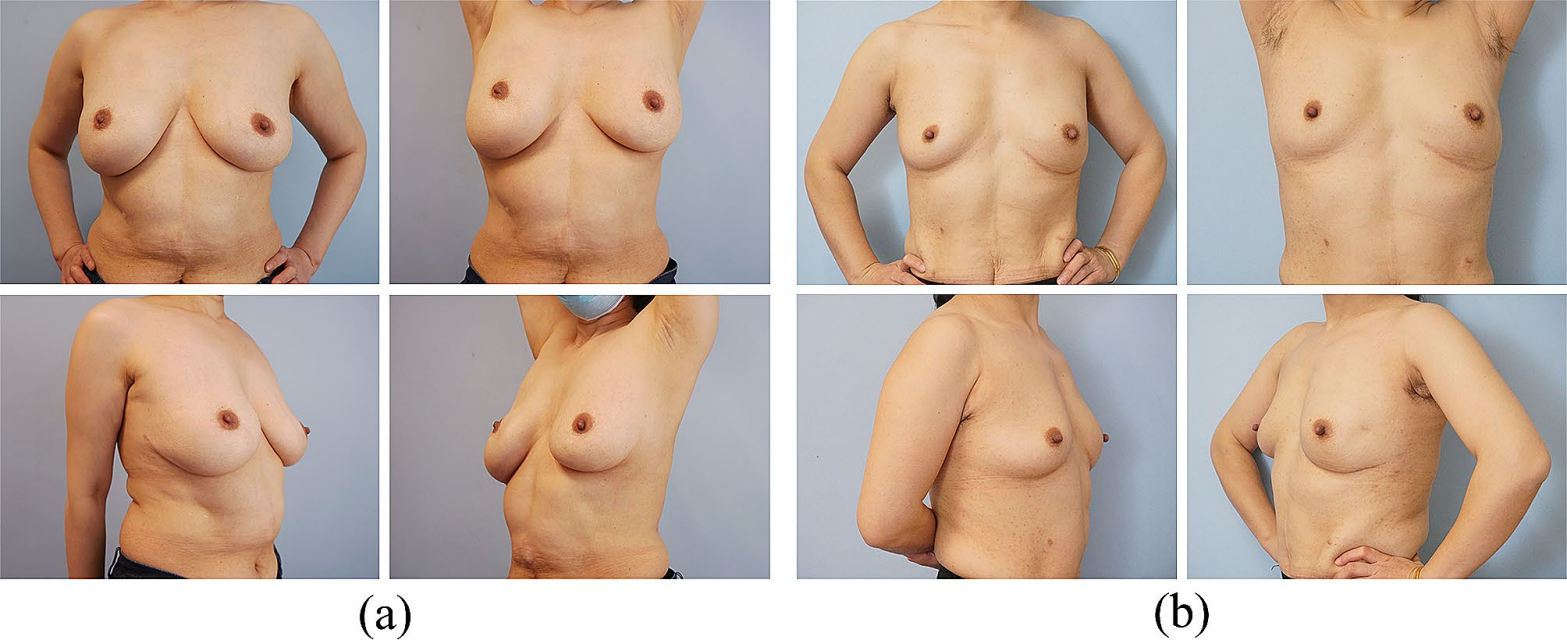
Cosmetic outcomes of immediate breast reconstruction using LHOF one year after surgery. (**a**) A 41-year-old patient with medium breasts underwent NSM and immediate reconstruction of the right breast. (**b**) A 47-year-old patient with small breasts underwent NSM and immediate reconstruction of the left breast. LHOF, laparoscopically harvested omental flap; NSM, nipple-sparing mastectomy

**Fig. 8:**
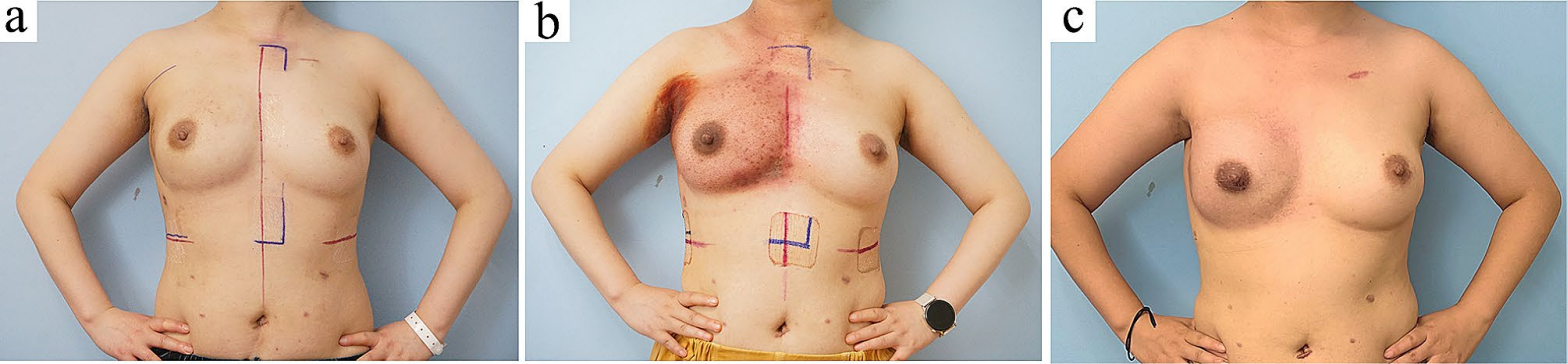
Reconstructed breast with LHOF appears insusceptible to radiotherapy. (**a**) Front view of a 38-year-old patient with LHOF reconstructed right breast before receiving radiotherapy. (**b**) Front view at 1 week after radiotherapy. (**c**) Front view at 1 year after radiotherapy. LHOF, laparoscopically harvested omental flap

The cosmetic outcomes were evaluated in 288 patients with a follow-up of at least 12 months (Table 4). Satisfactory cosmetic outcomes were observed in over 95% of patients by three-panel assessment and BCCT.core software. Soft and natural tactile feelings were the most outstanding features of breast reconstruction with the omental flap. The donor site scars were extremely tiny. All patients were satisfied with the minimal scars, and the inframammary fold incision was naturally hidden and made invisible. Representative postoperative images of the LHOF reconstruction are shown in Fig. 7. Radiotherapy had a less effect on the cosmetic result of the reconstructed breast using LHOF (Fig. 8).

**Table 4:** Cosmetic results (*n* = 288)

Cosmetic score	Panel assessment	BCCT.core, *n* (%)
Excellent	220 (76.4%)	151 (52.4%)
Good	54 (18.8%)	129 (44.8%)
Fair	9 (3.1%)	6 (2.1%)
Poor	5 (1.7%)	2 (0.7%)

BCCT.core: The Breast Cancer Conservative Treatment software

## Discussion

The omentum could be an ideal autologous flap for breast reconstruction, as it offers unique advantages compared to TRAM and LD flaps. The donor-site scars were minimal in the LHOF procedure. Moreover, TRAM and LD flap reconstruction necessitate a long incision at the donor site. The rate of complications associated with flap harvest was low in omental breast reconstruction. In this study, only one patient experienced a ventral hernia. However, over 20% of patients experienced complications such as hernia or postoperative bulge in TRAM flap reconstruction, with 12.7% of them requiring secondary surgery to repair abdominal wall weakness [3]. Some patients who underwent LD flap reconstruction were complicated with shoulder function impairment, muscle weakness, and seroma formation [2], thereby negatively impacting their quality of life. More importantly, volume loss was insignificant in reconstructed breasts when using the omental flap, which could potentially improve symmetry and enhance aesthetic results.

In the current study, the LHOF was performed by the same surgical team with extensive laparoscopic skills. The total operating time of the LHOF was considerably short, with a harvesting success rate of nearly 100%, with the exception of one pedicle injury. Patients with a history of abdominal surgery were not all excluded from LHOF in our study. Some patients had mild adhesion of the omentum to the abdominal wall or other organs due to previous surgery. Although the adhesions could be easily removed, LHOF reconstruction is not recommended for patients with multiple abdominal surgeries.

Subcutaneous fluid in the breast was the most common complication in the present study. Most of the patients reported swelling and pain in their reconstructed breasts. Fortunately, the effect resolved spontaneously within one week through drainage in most patients, and only a few cases required ultrasound-guided needle aspiration. The incidence of subcutaneous fluid accumulation ranged from 1 to 10% in previous studies [17, 18], while other small case series reported that no subcutaneous fluid was observed in omental breast reconstruction [24, 25]. The difference in subcutaneous fluid accumulation rates from other studies may be attributed to variations in sample size or patient selection. Despite the omentum having a high absorptive ability, subcutaneous fluid accumulation cannot be completely circumvented. In contrast, some patients may still develop hematoma or seroma in the early postoperative period. In our experience, patients with large breasts had a relatively higher risk of developing subcutaneous fluid accumulation, which could be due to an abnormal blood supply to the transferred pedicled omental tissue.

Necrosis of the omental flap is the most serious and concerning complication. In our study, partial flap necrosis occurred in 3.3% of the patients, which included one case of free omental flap. Notably, no total flap necrosis was found. Almost all cases of necrosis occurred during the early postoperative period and were mild and resolved spontaneously by conservative treatment. Zaha et al. reported that the incidence of partial necrosis was 5% in LHOF breast reconstruction [11]. Kim et al. reported a necrosis incidence of approximately 13% [15]. In a meta-analysis of omental breast reconstruction, the incidence of partial graft necrosis was 4.1% (17/410) [16]. Other studies reported that the incidence of partial necrosis in open omental flaps ranged from 2 to 16% [26–28]. Our findings were similar to the previous studies.Some causes of partial necrosis that have been identified are the following: (1) gastroepiploic hemal arch maybe undeveloped or absent due to anastomosis variations, resulting in a portion of the peripheral segments not receiving sufficient blood supply from a single pedicle. The hemal arch and branch vessels of the omental flap should be checked, particularly in the peripheral omentum tissue, and if a lack of blood supply is suspected, it should be trimmed. (2) In the process of moving it through the subcutaneous tunnel, trauma may injure some branch vessels. Especially, omental flaps of larger volumes are more challenging to extract through the subcutaneous tunnel. In such cases, repeatedly dragging the flap may cause injury to the branch vessels. (3) It may be compressed in the breast bed. (4) Adverse conditions (dehydration, hypothermia, trauma) may be present as the omentum is constantly exposed to the extraperitoneal environment. (5) Partial necrosis of the omental flap may be caused by accidental injury or resection of the epiploic branch vessels. During flap harvesting, epiploic branch vessels may be injured due to anatomical misidentification, especially in cases with a tight fusion between the anterior leaf of the transverse mesocolon and the posterior leaf of the gastrocolic ligament. In addition, severe necrosis of the omental flap can lead to reduced breast volume and negatively impact the aesthetic outcome. Therefore, LHOF requires careful dissection and gentle handling.

No severe complications related to LHOF occurred except for one case of pedicle injury and one case of ventral hernia. In contrast, hernia was a common complication in open omental flap harvesting [20]. However, the incidence of hernia has significantly decreased due to the use of laparoscopy [15–17] and remains a rare complication in LHOF. Pedicle injury was also more common in the early stages of this technique but became rarer as surgical skills improved.

In the present study, only a small number of reconstructed breasts exhibited a hard, stony shape, as previously reported in other studies [11, 19]. Breast hardness caused discomfort to patients in the early postoperative period. Changes in omental tissue, which may be caused by the change in blood supply, can result in breast hardness. In our experience, a large reconstructed breast was more likely to develop a firm texture. However, this effect was transient and the breast regained its natural softness within a few months without any intervention. The majority of patients were satisfied with the reconstructed breasts, while the main causes of cosmetic dissatisfaction were insufficient breast volume and breast asymmetry. Radiotherapy may cause temporary hyperpigmentation of the skin but exerts a lesser effect on the shape, size, and firmness of the reconstructed breast using LHOF. The omental flap demonstrated a relatively low sensitivity to irradiation. In the cases using implants combined with the pedicled omental flaps, no deformation or capsular contracture was observed after radiotherapy. The omentum has abundant vessels and stem cells. Theoretically, when radiation damage occurs, the omentum can produce angiogenic factors and growth factors, leading to a rise in blood vessel density, thus facilitating tissue regeneration, wound healing and injury repair [29, 30].

The main advantages of the omental flap include its rich vascularity, angiogenic capacity, great malleability, significant antimicrobial properties, and minimal donor-site morbidity. The great malleability allows natural ptosis of the reconstructed breast, matching the contralateral breast. Additionally, using an implant in breast reconstruction increases the risk of infection. Yet, patients in this study receiving LHOF combined with implants did not develop local infections, which may be partially attributed to the antimicrobial properties of the omentum. However, more evidence is needed to confirm this correlation.

Nevertheless, volume insufficiency of the omentum remains a disadvantage for autologous breast reconstruction, and there is no effective method to accurately estimate the omentum volume before surgery. Diagnostic laparoscopy can be performed to evaluate the omentum volume, but this invasive examination is not acceptable to most patients. Hence, the patients were counseled prior to the surgery that implants might be used if the omental volume was insufficient. In this study, insufficient omental volume occurred in 34% of the patients. In appropriately selected patients, the omental flap is fully suitable for total breast reconstruction. Even in patients using implants, the omental flap could also help improve the tactile feeling of reconstructed breasts.

The oncological safety of the omental flap remains a major concern in oncoplastic breast surgery [20]. Theoretically, stem cells with neovascularization potential and potential oncogenic factors from fat cells are risk factors for tumor recurrence [31, 32]. However, clinical evidence does not support this theory. A systematic review of omental flap reconstruction reported a very low tumor recurrence rate [16]. In our study, three patients developed tumor recurrence, showing a similar recurrence rate to previous studies [15], indicating the oncological safety of LHOF breast reconstruction.

To the best of our knowledge, this is the largest study on immediate breast reconstruction using LHOF. However, the limitations of this study should be acknowledged. First, the study was a single-center analysis. Second, the follow-up time was relatively short. Long-term follow-up studies are required to further identify the cosmetic outcomes and oncologic safety of LHOF breast reconstruction. Third, this was a retrospective study with no comparison group. In the future, prospective controlled studies are warranted to provide solid evidence for LHOF breast reconstruction.

## Conclusions

LHOF appears to be a safe and feasible option for immediate breast reconstruction, involving minimal donor-site morbidity and deformity, also providing satisfactory cosmetic results and promising oncologic outcomes.

## Data Availability

No datasets were generated or analysed during the current study.

## Abbreviations

NSM: Nipple-sparing mastectomy
BCS: Breast-conserving surgery
LHOF: Laparoscopically harvested omental flap
LD flap: Latissimus dorsi myocutaneous flap
TRAM flap: Transverse rectus abdominis myocutaneous flap
BCCT: Breast Cancer Conservative Treatment
NAC: Nipple areola complex
BMI: Body mass index
PET/CT: Positron emission tomography/computed tomography
MRI: Magnetic resonance imaging
